# Counter a Drone in a Complex Neighborhood Area by Deep Reinforcement Learning

**DOI:** 10.3390/s20082320

**Published:** 2020-04-18

**Authors:** Ender Çetin, Cristina Barrado, Enric Pastor

**Affiliations:** 1 Aerospace Science and Technology, UPC BarcelonaTECH, 08860 Castelldefels, Spain; 2 Computer Architecture Department, UPC BarcelonaTECH, 08860 Castelldefels, Spain

**Keywords:** counter drones, UAV, deep reinforcement learning, transfer learning, double deep Q-network (DDQN), joint neural network (JNN)

## Abstract

Counter-drone technology by using artificial intelligence (AI) is an emerging technology and it is rapidly developing. Considering the recent advances in AI, counter-drone systems with AI can be very accurate and efficient to fight against drones. The time required to engage with the target can be less than other methods based on human intervention, such as bringing down a malicious drone by a machine-gun. Also, AI can identify and classify the target with a high precision in order to prevent a false interdiction with the targeted object. We believe that counter-drone technology with AI will bring important advantages to the threats coming from some drones and will help the skies to become safer and more secure. In this study, a deep reinforcement learning (DRL) architecture is proposed to counter a drone with another drone, the learning drone, which will autonomously avoid all kind of obstacles inside a suburban neighborhood environment. The environment in a simulator that has stationary obstacles such as trees, cables, parked cars, and houses. In addition, another non-malicious third drone, acting as moving obstacle inside the environment was also included. In this way, the learning drone is trained to detect stationary and moving obstacles, and to counter and catch the target drone without crashing with any other obstacle inside the neighborhood. The learning drone has a front camera and it can capture continuously depth images. Every depth image is part of the state used in DRL architecture. There are also scalar state parameters such as velocities, distances to the target, distances to some defined geofences and track, and elevation angles. The state image and scalars are processed by a neural network that joints the two state parts into a unique flow. Moreover, transfer learning is tested by using the weights of the first full-trained model. With transfer learning, one of the best jump-starts achieved higher mean rewards (close to 35 more) at the beginning of training. Transfer learning also shows that the number of crashes during training can be reduced, with a total number of crashed episodes reduced by 65%, when all ground obstacles are included.

## 1. Introduction

According to the European Air Traffic Management (ATM) master plan drone roadmap [1], the unmanned aerial Vehicles (UAV), also known as drones, will scale their flights and their operations beyond visual line of sight (BVLOS) will cover most of the air traffic by 2035. Internet of Things (IoT) has shown to be very adequate for dealing with such growth, for instance by enabling distributed approaches for collision avoidance [2]. However, this increase on the number of drones in the airspace worldwide attracts people who can misuse the UAVs. Under the latest drone regulation it is stated that drones should not enter restricted zones, such as airports, but there is no clear countering drone solutions yet. In 2018, a drone caused a huge problem in Gatwick London Airport. The flights were canceled and around 140,000 passengers were affected [3]. The number of drones will increase and it is obvious that they can cause more serious problems. The threat is coming and counter measurements should be taken.

There are many study cases which investigate countering drones. In a study presented in defence science journal [4], UAV detection and elimination are discussed. The terminology used in this journal is based on The North Atlantic Treaty Organization (NATO). According to this terminology, the problem of defence is divided into 3 main aspects: Air surveillance, command and control and elimination. Air surveillance is used for detecting and identifying UAV. Command and control collects data from sensors and acts as a decision maker mechanism when the actions are taken against the aerial object. Elimination is a collection of methods for interdiction of the threats. Devices to detect UAV, such as radar systems, sensors or acoustic devices, are existing technologies used for countering drones. To eliminate these threats several methods are proposed. These methods include shotguns, laser guns, nets, water cannons, birds trained for catching drones, jamming the command and control radio signals and jamming the global navigation satellite system signals. In Table 1, counter-drone methods and their number of cases available now are presented.

In recent years, researchers proposed some studies in the area of deep reinforcement learning (DRL) and UAV. In this context, the studies are mostly focused on the topics of drone detection and of navigation of drones in an unknown environment, avoiding obstacles.

Akhloufi et al. [6] propose deep reinforcement learning and deep search areas for drones’ pursuit-evasion problems. Firstly, DRL is used to follow a target drone, by predicting its actions to follow the target. Also, supervised learning is applied by using a large dataset of drone images. Another example is to predict the position of the target drone using deep object detector and search area proposal. YOLO v2 [7] is used as an object detector.

Anwar et al. [8] studied the DRL for autonomous navigation. Transfer learning is applied to reduce the training computation load. The environment is designed in Unreal Engine [9] and tested in the real world, by using a low-cost drone (a DJI Tello), with the similar results obtained. This research code was published as open source and contains the comparisons between the real environment results and the simulation results.

In another research by Kouris et al. [10], it was proposed that a self-supervised Convolutional Neural Network (CNN)-based approach can be used to navigate a drone autonomously and to avoid collisions. A regression CNN is used to predict the distances between the agent and the obstacles. The distances to the closest obstacle in different directions are estimated. The drone flight parameters, such as the linear velocity and the yaw angle of the drone, are changed according to the predictions made through the deep neural network.

DRL is also used against jamming. In a research by Lu et al. [11] DRL methods were applied to choose the relay policy by using a drone as part of a cellular communication framework against jamming. In this method, the cellular systems can resist the jamming without knowing the jamming model and the network model. In this article, it is stated that the optimal performance can be achieved by adequately interacting with the jammer.

Rodriguez-Ramos et al. [12] proposed a DRL for the autonomous landing of drones on a moving platform. The drone control during landing is performed using the deep deterministic policy gradients (DDPG) algorithm and tested over a simulator interface.

In this paper, deep reinforcement learning is proposed to train a drone to counter another drone. The learning drone is the DRL agent and is trained to counter the target drone by crashing against it, but it is also trained to avoid any other obstacle. The remainder of the paper is organized as follows. In Section 2, the theory about deep reinforcement learning and the basics of transfer learning metrics are provided. Section 3 provides tools and methods, this is, the DRL model which includes the environment, the states, the actions set and the reward function used in the DRL algorithm. In Section 4, the performance of several training cases and their results are presented. In Section 5, some further details of the models and their results are analyzed and discussed. Finally, in Section 6, conclusions and the future work are presented.

## 2. Methods

**Reinforcement learning** (RL) is an approach to AI based on trial and error experiences by interacting with the *environment*. In RL, the *agent*, or learner, can make a decision and take an action which updates the environment. Environment state is updated by each action that the agent takes. Also after each action the environment outputs a reward, in the form of a scalar value. Rewards reveal information about an action, whether that action results in positive or negative feedback. The objective of the agent is to maximize the cumulative reward. Each iteration between an action and the next action is known as step. An action can lead the environment to a terminal state, which is also known as the end of an episode. Thus, an episode is the set of steps starting at an initial state and finishing with a terminal state.

**Figure 1:**
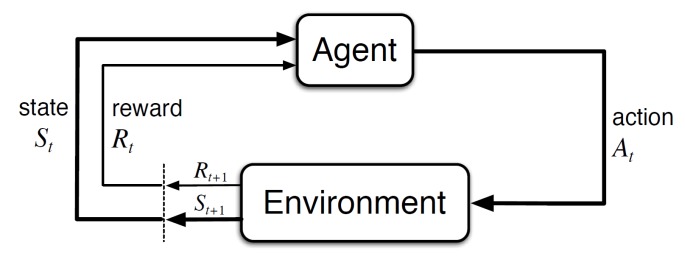
The agent-environment interaction in reinforcement learning [13].

In this paper, the agent is a quadcopter drone that is rewarded in each step by the environment. A collision of the drone always ends the episode. Also a timeout of 300 s is set to end the episode. The actions executed by the agent are the inputs to the environment and the states and rewards are the outputs of the environment as shown in Figure 1. *State* is represented as $S_{t}$ and the *State Space* is represented as *S*. The interaction between the agent and the environment is in discrete time steps *t*. *Action* and *Action Space* are represented as $A_{t}$ and $A(S_{t})$ respectively. Reward values are updated in each time, $R_{t+1}$, and a new state becomes $S_{t+1}$.

In RL, states are mapped with the probabilities of the next rewards for the possible actions in each time step *t* and it is called *policy*. In RL, the policy is chosen to maximize the cumulative reward over time. The fundamental concepts for RL are explained in detail in surveys [13,14].

As a particular case of RL, the use of a (deep) neural networks to build the decision making algorithm of the agent, is known as deep reinforcement learning (DRL). In a research by Mnih, V. et al., it is shown that DRL algorithms can beat human performance level in video and board games [15]. Several implementations of DRL, such as deep Q-network (DQN) [16] or double deep Q-network (DDQN) [17] are being proposed and showed improved results. The main goal of DQN is to use a deep convolutional neural network to approximate the optimal action-value function. DQN provides updating action values and target values iteratively. Moreover, it proposes the experience replay, which randomizes the data and improves the data distribution. Experience replay is demonstrated and explained in several research works [18,19,20]. The DDQN method, initially proposed in [21], is basically decoupling the action selection from the evaluation. Although one estimate is updated per step, two estimates are learned in a random selection. DQN and Double DQN algorithms were previously tested for drones learning in our simulator environment [22], showing that both algorithms were successful to reach a fixed destination. In this work the authors have decided to use DDQN as this algorithm showed the best performance among those tested.

Deep reinforcement learning is capable of handling difficult complex problems. However, learning can be too slow or even infeasible. For this reason, researchers in DRL have focused on improving the time spent on learning by implementing various approaches. The most successful is **transfer learning** (TL). The main purpose of TL is to improve the learning performance by using the experience from successfully pre-trained models [23].

**Figure 2:**
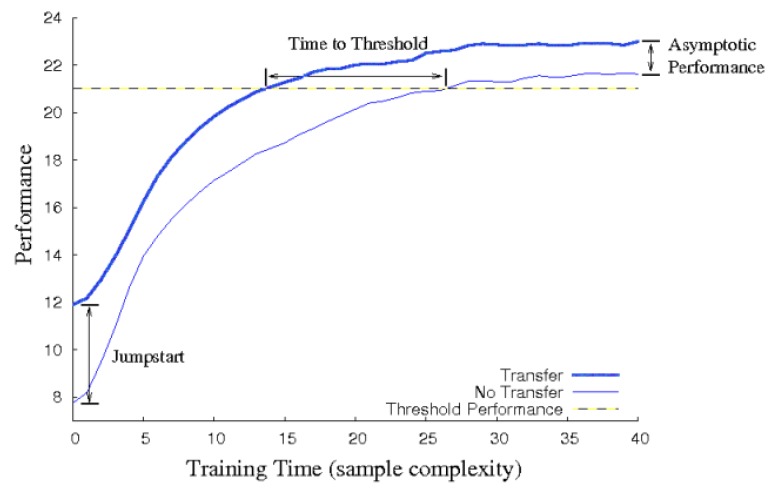
Different metrics for measuring TL [23].

Transfer learning can be used for different goals and in different situations. Several evaluation metrics can be addressed in order to evaluate the TL algorithms. In Figure 2, common parameters for measuring TL performance are shown. The difference between the initial reward values, with and without TL, is called jump-start. The final performance of the agent is named as asymptotic performance and the time required to achieve a pre-defined level is called time to Threshold. These metrics are explained in detail in [23].

## 3. DRL Model Definition

### 3.1. Tools

This section presents the tools and tool-kits used for developing, training and comparing the proposed DRL algorithms: the AirSim simulator, OpenAI-Gym and Keras-RL.

**Figure 3:**
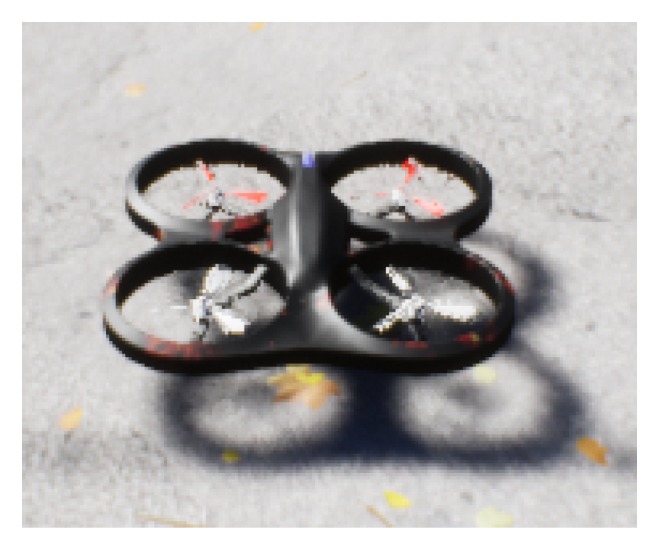
Quadcopter used in AirSim.

AirSim is a platform for AI research to experiment with deep learning, computer vision and reinforcement learning algorithms for autonomous vehicles [24] such as cars or drones. AirSim it is built as a Unreal Engine [9] plugin. Unreal Engine provides ultra realistic rendering and strong graphic features for the Airsim. The quadcopter used in the Airsim simulator can be seen in Figure 3.

AirSim has many environments available to be used in reinforcement learning. These environments are mountains, blocks, cities, etc.

It is important to mention that AirSim is not deterministic. The simulator has its own physics and dynamics, which can be affected by simulated environmental conditions, such as wind, but it is also affected by some random white noise. For this reason, as in real life, the same actions give not exactly the same response when applied in different simulations.

OpenAI-Gym [25] is an open source interface to reinforcement learning tasks. It is a toolkit for developing and comparing reinforcement learning algorithms. It is compatible with most common neural networks tools, such as Tensor Flow [26] or Theano [27]. The gym library has a collection of environments to test reinforcement learning algorithms. These environments have a shared interface which allows writing general algorithms.

Keras-RL [28] implements some state-of-the art deep reinforcement learning algorithms in Python and seamlessly integrates with the deep learning library Keras. Keras can work with OpenAI Gym and is built according to the developer needs, giving the ability to define own callbacks and metrics.

### 3.2. Model

This section presents the decisions taken when building the DRL model. These decisions include: The selected data to capture from the environment and how these data need to be processed to create the state of the agent; The architecture of the neural network used to process such state and to return the action with the most expected reward; The set of actions available to the drone; And the reward function formulation.

#### 3.2.1. Environment

**Figure 4:**
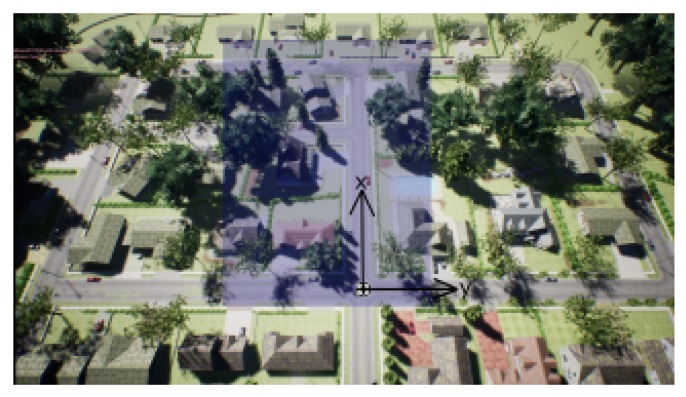
The neighbourhood environment.

From the number of AirSim environments available for AI research to experiment with deep reinforcement learning algorithms, we decided to select a small urban neighbourhood. The reason is that currently some drones operators are starting to operate in similar environments, which may be used by malicious drones to enter the area too, and to become a thread for its inhabitants. A counter-drone system is needed to avoid this not desired incomers. The tested neighborhood environment is shown in Figure 4. A two-dimensional representation of the environment is shown by using the x and y axis at the origin point of the agent. The agent starts at (0,0,0) position in the NED coordinate system.

We assume that the counter-drone system must remain always within the neighbourhood that has contracted it. The limits of the area of the contract are given in the form of a geofence [29], which limits are shown in shaded blue region of Figure 4. A geofence is defined as a technology that creates a virtual barrier around a geographical area. The geofence technology is used in drone navigation in order to create constraints for drones, with the purpose to keep the drones within the predefined area (also known as geocage, as in our proposed environment) or out of it. For instance, in a study about a generic and modular geofencing strategy for civilian drones [30], geofence is proposed as a means to avoid entering controlled airspace areas and even to avoid collisions with the environment, people, or other flying vehicles. If the geofenced area is violated, the operator and/or the authorities can be notified and actions can be taken to prevent further incursions into the area.

#### 3.2.2. State Representation

The state of the model is composed by an image and several auxiliary scalar values:

- Image

**Figure 5:**
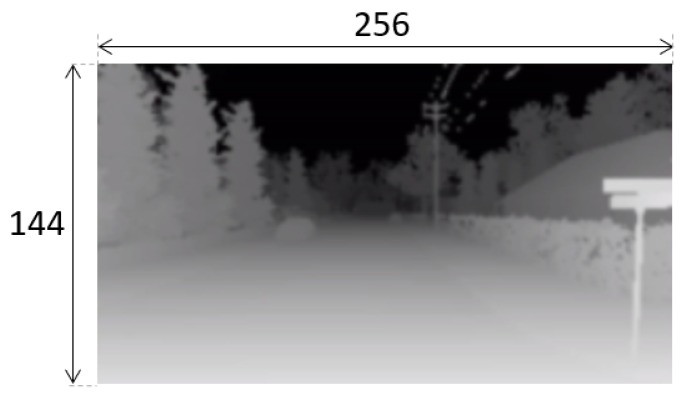
The Depth Image.

**Figure 6:**
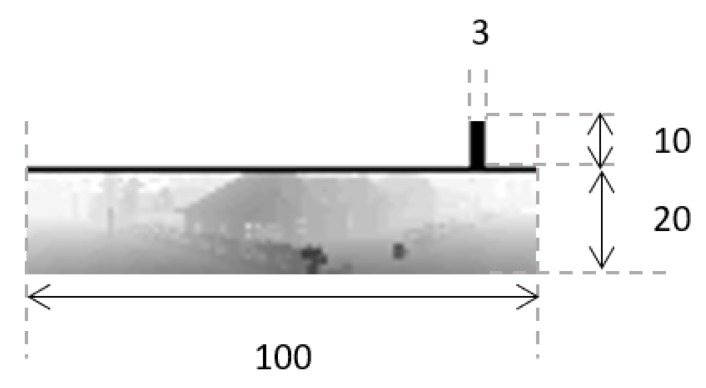
The State Image and Encoded Section.

Most drones have one or more cameras facing front, able to capture the objects situated in the flying direction. AirSim provides three virtual front cameras: visual, thermal, and depth. For the state we selected the image received from the depth camera. According to the AirSim [31], the depth camera output is received by using Airsim API “Depth Perspective” image type, which simulates the return of a projection ray that hits its pixels. The depth image received from the camera is a 256 × 144 pixels image as shown in Figure 5. From this image we crop a central part and create the state image. This state image is set as 30 × 100 pixels and is shown in Figure 6. The bottom of the image includes the cropped central part of the depth image (20 × 100 pixels). Then, the top 10 rows of the state image is white (no obstacles), except for the 3 × 10 pixels black line. This black line is used to represent the track angle, this is, the suitable direction to find the target drone [22]. This 3 × 10 pixels black line moves left and right according to the relative movements of the target and the catching drones.

**Figure 7:**
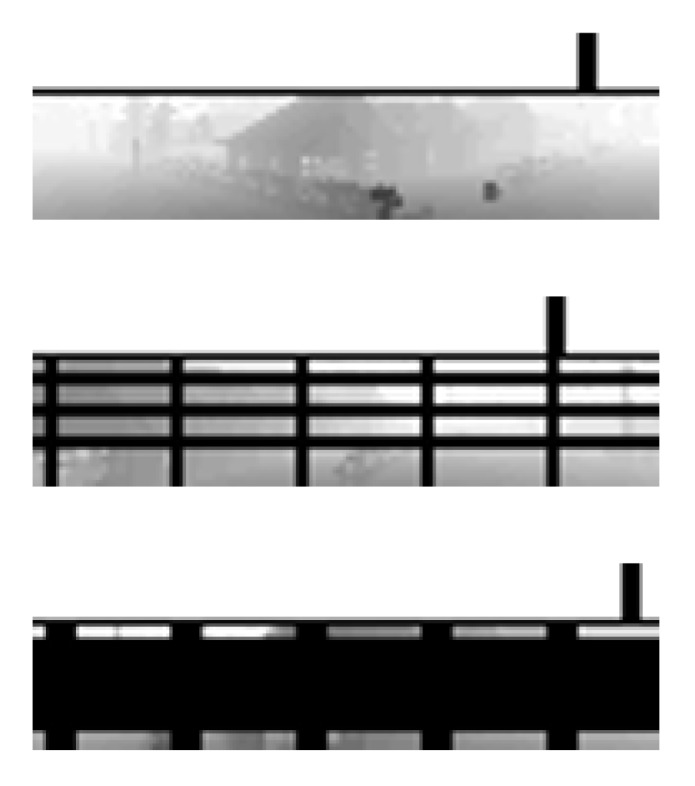
Fences drawn on the State Image.

Additionally, as Figure 7 shows, a grid is drawn on top of the state image when the drone is close to cross the geofence. The thickness of the grid increases as the drone moves towards the geofence limits. The grid appear when the separation distance between the drone and the geofence limits becomes lower or equal to 4 m.

- Auxiliary Inputs

Additionally to the images, AirSim provides, though its application programming interface, the capability to retrieve data of the environment. For example, data provided by AirSim includes the Euler angles, the position and the orientation of any drone flying in the environment, their linear and angular velocities, and also the linear and angular accelerations. For the purposes of our agent we selected as relevant the following data: the Euclidean distance, the track angle and the elevation angle of the target drone from the current position of the training drone. Other auxiliary data selected as relevant for the state is the distances to geofence limits.

The summarize, the auxiliary data, aggregated to the state image as part of the agent state, is:

- The velocity of the agent in *x* and *y* directions: $v_{x}v_{y}$
- The distance from the agent to the goal in *x* and *y* directions and the Euclidean distance: $d_{x}d_{y}d_{t}$
- Track and the elevation angles between the agent and the goal: ψζ
- The distances to the four geofence limits

#### 3.2.3. Agent’s Neural Network

**Figure 8:**
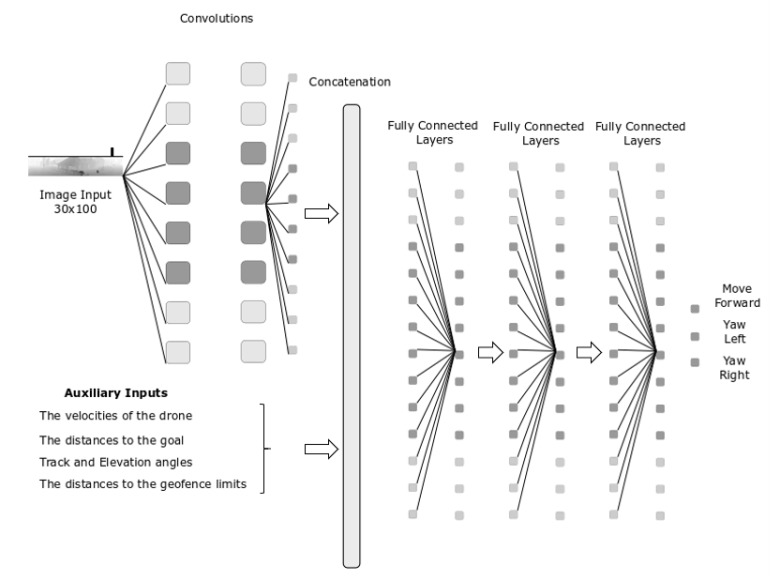
The Agent.

The full state, composed by the image and the auxiliary data, is processed with a neural network, which architecture is shown in Figure 8. The image is the input of a convolutional neural network (CNN), followed by a flatten layer. Then a concatenation layer joints the flatten output of the CNN with the scalar auxiliary data of the state.

**Figure A1:**
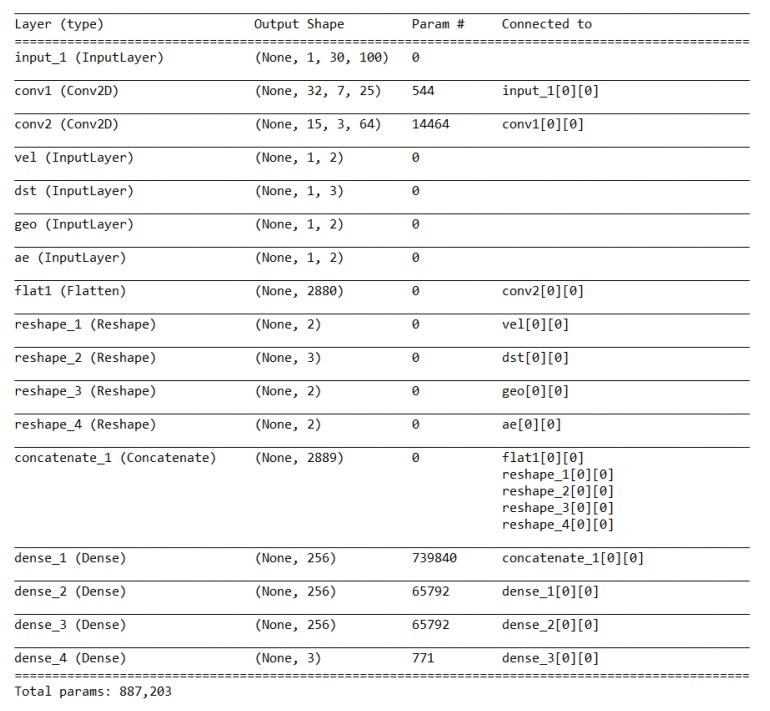
Neural Network Model Summary.

The first layer of the CNN consists of RELU activated 32 kernel 4 × 4 with stride 4. This layer is followed by RELU activated 64 kernel 3 × 3 with stride 2. The output of the sequential CNN model is concatenated with the reshaped scalar values and the concatenated tensor becomes the input of three RELU activated consecutive 256 kernel dense layers. The output layer is a dense layer and the outputs are the action values. Neural network model summary can be seen in Figure 8 and with more detail about the layers and their parameters in the Appendix A Figure A1.

#### 3.2.4. Actions

In this paper, the drone agent is flying always in the same plane in which the target drone is found, this is, without changing altitude during the training. To move the learning drone we have selected, from AirSim available interfaces to control vehicles autonomously, the following three simple options:

- Straight: Straight movement in direction of the heading with speed equal to 4 m/s
- Yaw left: Rotate clockwise around z axis with a 30 deg/s angular speed
- Yaw right: Rotate counter-clockwise around z axis with a 30 deg/s angular speed

**Figure 9:**
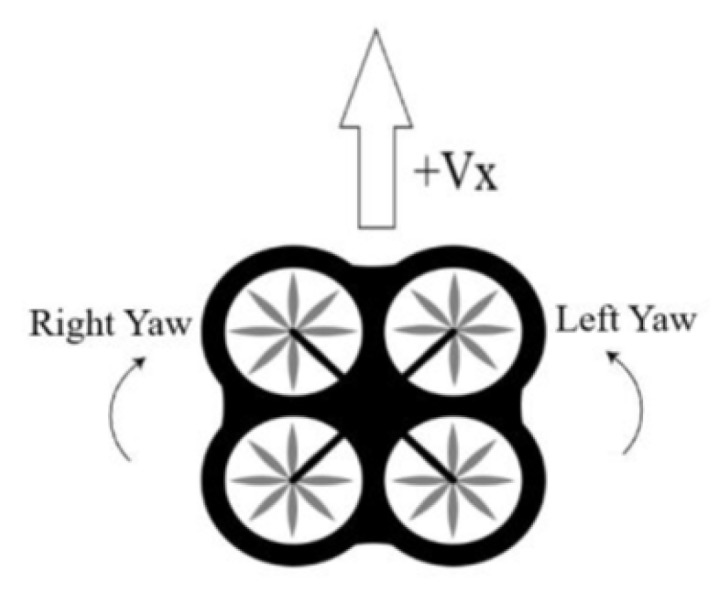
Action Space.

Figure 9 shows the representation of the three actions.

As a consequence, the output layer of the agent’s neural network consists of three activation values, one for each possible action. The neural network will predict which of the three actions has a higher probability of obtaining the maximum cumulative reward.

#### 3.2.5. Rewards

The proposed reward function is shown in Table 2. At the end of an episode the agent is rewarded +100 if the episode is successful, this is, it has ended by catching the target drone. On the other hand, the agent is penalized and it is given a reward −100 if the episode was unsuccessful, this is, it has ended because the agent had a collision with a visible obstacle of the environment or because had violated the geofence. Additionally, every intermediate step returns a reward of −1 to penalize delays on achieving the agent’s objective. The reward of an intermediate step has two bonus: plus ΔDistance, which is distance-to-the-goal reduction with respect to the previous step, and plus trackangle which represents the zero-deviation towards the target direction.

## 4. Training Analysis & Results

### 4.1. Training Setup

The proposed DRL model is trained on a desktop with NVIDIA GeForce GTX 1060 with 6 GB RAM graphic co-processor and Intel i7 processor, 16 GB of memory. A full training phase, for instance, the case named below as Baseline, lasted for 125,000 steps, and spent around 48 to 56 h. The Adam optimizer is used in the feed backward of the neural network. The same resources are used in both training and testing. The tests, run much faster, and are used to evaluate the learned capabilities of the agent training.

**Figure 10:**
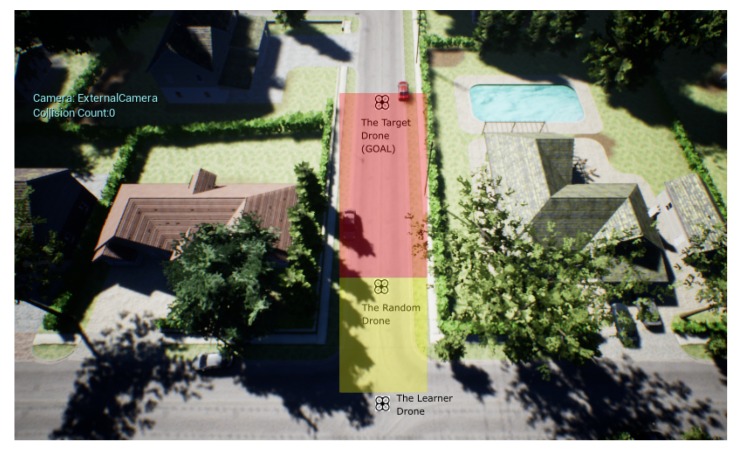
The environment with Random Drone, Target drone and the Learner Drone.

Figure 10 shows the simulation setup, with the starting location of the three involved drones: The agent, also known as learning drone, in in the bottom of the image; On the top of the image we find the target drone, this is, the malicious drone that the agent has to catch; In between both some simulations include a third drone, named as random drone. This is used as a moving obstacle that the agent shall avoid. The target and the random drones move randomly from their starting point, inside the shaded areas of Figure 10: red for the target drone (sized 25 × 8 m), yellow for the random drone (sized 10 × 8 m). Target drone and random drone can change positions up to 1 m in each step.

The learning drone is always started in the same (0,0,0) location, but its yaw angle is random. This aims to increase the exploration capabilities of the learning drone from the first step.

### 4.2. Definition of Cases

Several training experiments are defined and categorized into two groups. The first group of training experiments are performed at 30 m height, above trees, houses and cars. In this first group the only obstacles in the environment are the geofence, the random drone and the target drone. The second group of training experiments are performed at low altitude, at an altitude of four meters. The main reason is that the drone can interact with all kind of obstacles such as trees, houses, and electrical wires found at this level. More detail about the obstacles in the environment can be seen in videos from youtube (https://www.youtube.com/watch?v=wFDGZANAcfQ&feature=youtu.be). In each group of training, cases with and without transfer learning are implemented to analyze and compare the performance of the models. All transfer learning models use a unique pre-trained model. The pre-trained model is built in the first presented case and named Baseline.

Table 3 summarizes the different cases explained through the section.

### 4.3. Training Results

In this section the performance of the training cases are analyzed.

#### 4.3.1. Case 1: Training at 30 m Height

The following figures show the training performance relating the number of step (in the x-axis) with its cumulative reward (in the y-axis). The light blue represents the actual reward value of the step and the dark blue represents the mean rewards of the every 100 steps. The time steps are discrete and equal to one second. The vertical dotted line represents the end of the annealing training part. There is a linear epsilon-greedy exploration before the annealing points, starting from full random down to 10% random. After the annealing point, the 10% random is maintained until the end of the training. There is no random during the exploitation (tests).

The training episodes are finished when the drone catches the target or when the learning drone collides with any stationary or non-stationary obstacle (random drone) or when the learning drone overpasses the geofence limits.

- Baseline: Training including geofence and target drone

**Figure 11:**
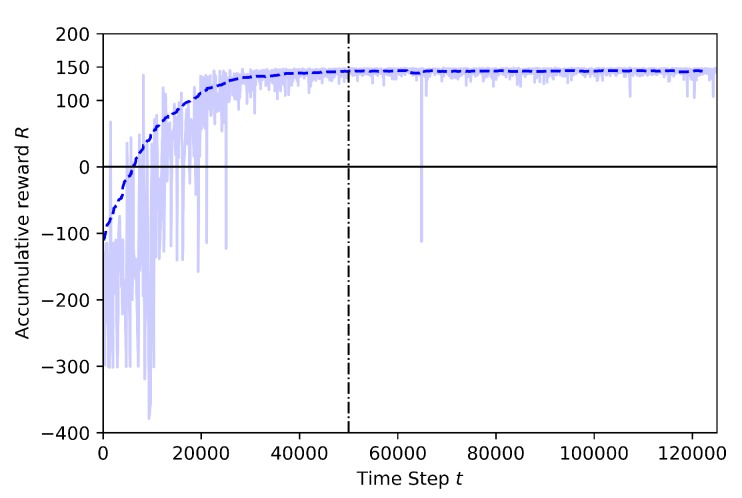
Training result for Baseline.

In Figure 11, the training results for 125K steps are shown. This training is set at 30 m altitude, where both the learner drone and target drone are flying. This training is known as “Baseline” because it is used as a pre-trained model for training by transfer learning which will be explained in the next cases. At the beginning of the training, the learner drone explores the environment and the rewards are mostly around −300 which is a very low reward. The main reason is that at the beginning of the training the random behavior is very high and the drone has not yet knowledge of how to catch the target, thus, it exceeds the episode time limit. However, the learning curve sets a higher slope at 20K steps and the cumulative reward reaches the highest values around 40K steps, before the annealing point. Although there are 61 episodes crashed during training, there are no crashed episodes happened after annealing. This training shows that after a certain time the drone learns how to avoid geofence limits and how to catch the target as soon as possible.

- Case 1.1: adding a stationary third drone

**Figure 12:**
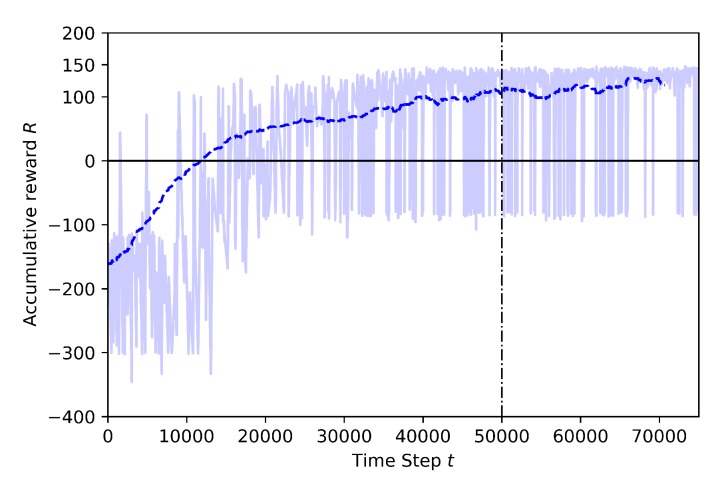
Training result for Case 1.1.

This training is set at 30 m height with a stationary drone is placed in the environment at this same altitude, same as the learner drone and target drone. In Figure 12 the full training results for 75K steps can be seen. At the beginning of the training, the learner drone explores the environment as it is seen in the previous training and the rewards are mostly around −300. The main reason is the same as before, a very high initial random behavior. However, this case has the stationary drone placed just 5 m away from the learning drone. As a result, it is observed that the behavior of the learning drone is different than the training shown in Figure 11. There are more crashed episodes in this training because of the stationary drone placed in the environment. The learning drone eventually learns how to avoid this stationary drone but it takes a while. For example, the number of crashes against the random drone decreases after 65K steps. The cumulative reward reaches the highest values around 70K steps.

- Case 1.2: adding a stationary third drone and using pre-trained model from Baseline

**Figure 13:**
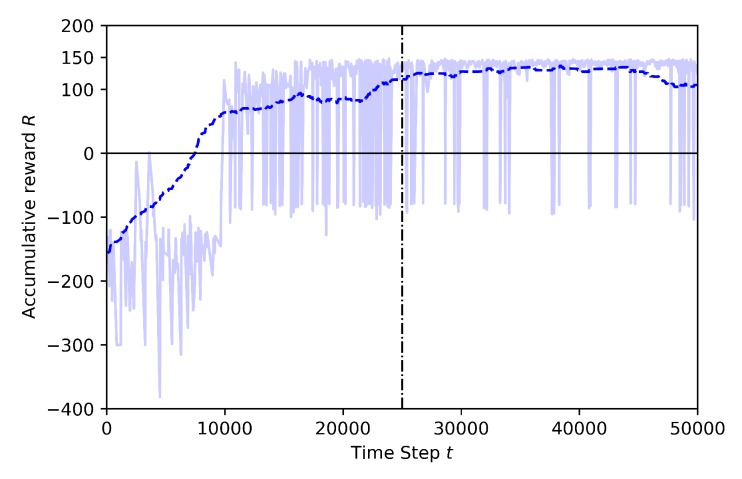
Training result for Case 1.2 by Transfer Learning.

In Case 1.2 the training is performed by using transfer learning. In this training the Baseline pre-trained model, which is shown in Figure 11, is already trained to catch the target while avoiding the geofences, and the new training focus only on the new knowledge: the avoidance of the stationary drone. The training time is finished after 50K steps and annealing point is set at 25K steps in this training. The last layer of the model is frozen and the other layers are trained. In Figure 13 the training results for transfer learning are shown. As it is seen in this figure, at the beginning of the training, the cumulative reward reaches positive values very fast, if compared to the training seen in Figure 12. There are still some crashes but these are caused by the stationary drone and the high random behavior of the learning drone before the annealing point. After the annealing point, the learning drone is in general able to catch the target drone and to successfully avoid the stationary drone and the geofences.

**Figure 14:**
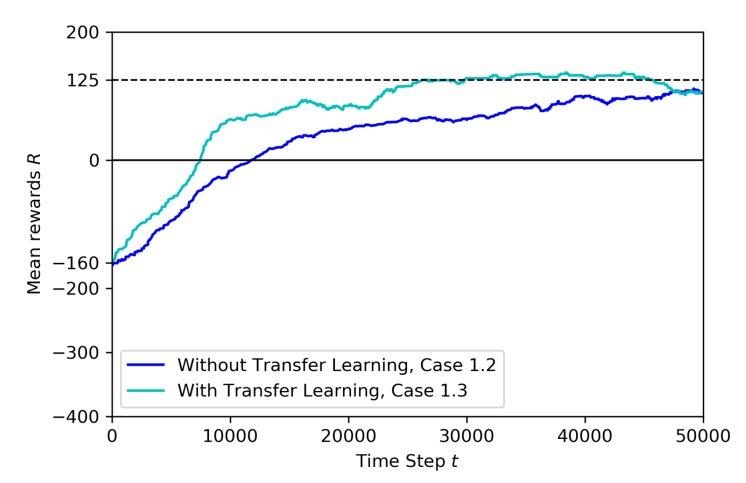
Training mean rewards for Case 1.1 and Case 1.2.

Figure 14 shows the transfer learning metrics: there is no jump-start, but the threshold time can be observed. Both training start their curve with −160 mean reward. However, during the training with TL, the agent reaches a pre-specified performance level faster (at around 30K steps) than the model without TL. The asymptotic performance level is zero at the end of 50K steps.

- Case 1.3: adding movement to the third drone

**Figure 15:**
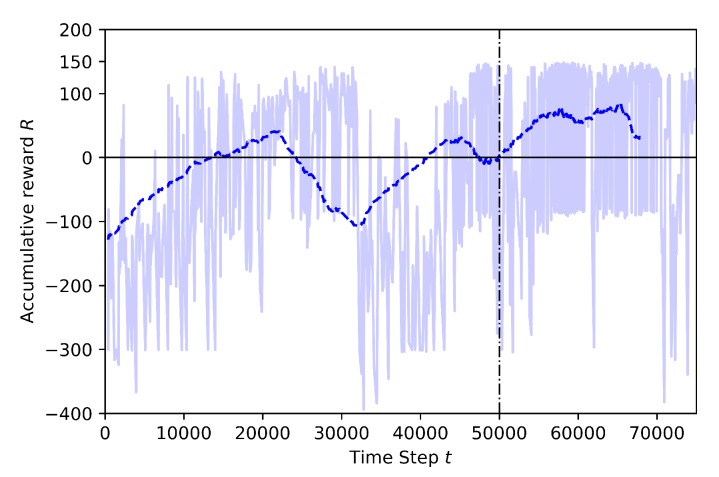
Training result for Case 1.3.

In this case, a third drone moving randomly is placed into the environment, at the same altitude than the learner drone and target drone. In Figure 15 the training results for 75K steps can be seen. At the beginning of the training, the rewards are mostly around −300 because the learning drone still explores the environment. It is observed how the behavior of the drone changes before and after identifying the non-stationary third drone. Before, the mean rewards curve goes up until 20K steps since the drone seems to learn how to avoid geofence and the non-stationary drone at the beginning, but after the curve starts going down for a while and the rewards are mostly around −300 which are mostly considered time-limit. The main reason is that the drone starts exploring again until catching the target drone. After annealing, the mean rewards looks stable, but there are still episodes that crash because of the non-stationary drone moving randomly.

- Case 1.4: adding movement to the third drone and using pre-trained model from Baseline

**Figure 16:**
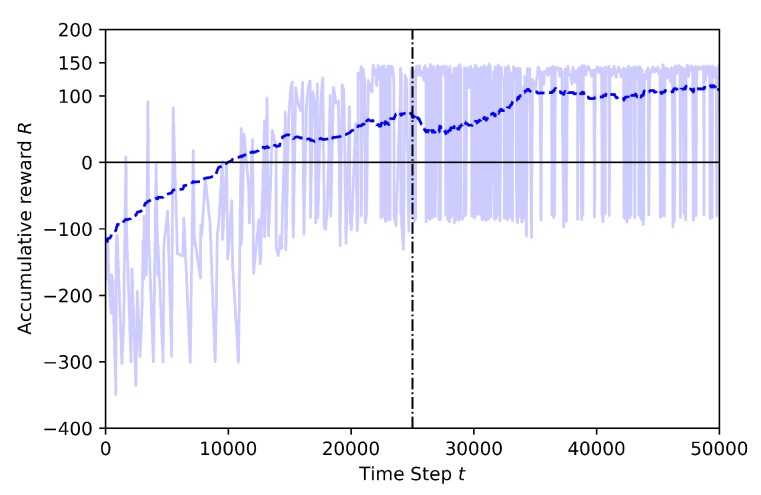
Training result for Case 1.4 by Transfer Learning.

In this training, the Baseline model, which is shown in Figure 11, is used as the pre-trained model of transfer learning, to train the learning drone to avoid geofences and the non-stationary drone and to catch the target drone. The last layer of the model is frozen and the other layers are trained. The training time is 50K steps and annealing point is at 25K steps. In Figure 16, the training result for transfer learning is shown.

At the beginning of the training, the cumulative rewards start at −115 and but reach high values after the annealing point. The cumulative reward is more stable during the training with TL compared to the training seen in Figure 15. This is because the crashes caused by the non-stationary drone after annealing point in the second case. Thanks to transfer learning, the number of crashes with the geofence are reduced by almost 75%. The non-stationary drone is a hard challenge for the agent, because it can hit the agent during the training and thus the learning takes longer.

**Figure 17:**
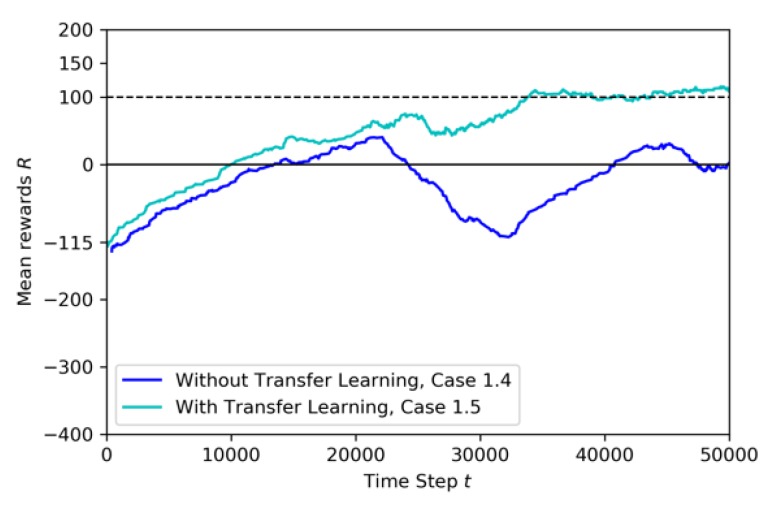
Training mean rewards for Case 1.3 and Case 1.4.

Figure 17 compares the reward curves for training with and without the transfer learning. Training without transfer learning is not smooth. Both curves start the training with mean reward around −115. However, with TL, the agent reaches a pre-specified performance level, which is at 100 mean reward, and does it faster (at around 35K steps), as expected. On the contrary, without TL the curve is not stable, with many up and downs caused by the unexpected random movement of the non-stationary drone. Since the target drone have the same image and also moves randomly, the situation creates confusion and the full training is not successful.

#### 4.3.2. Case 2: Training at Low Altitude, with Many Obstacles

- Case 2.1: without Transfer Learning

**Figure 18:**
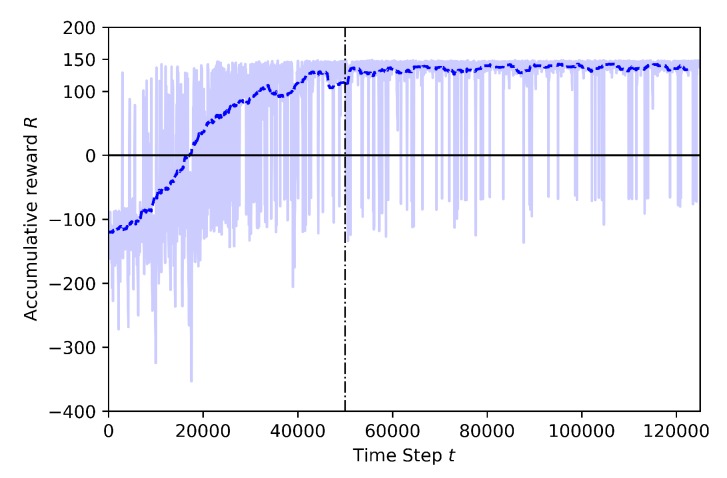
Training result for Case 2.1.

In Figure 18 the results for 125K steps full training are shown. The training is set at 4 m altitude and in addition to the obstacles, such as trees, houses, power cables and cars, the learning drone and target drone are added to the environment. At the beginning of the training, the learning drone explores the environment and the rewards are mostly around −300. This is a very low reward during all training session in this case. However, the learning curve goes up after 25K steps and the cumulative reward reaches higher values around 35K steps, before the annealing point. This training shows that after a certain time, the drone learns how to avoid all kind of obstacles including geofence limits, and how to catch the target as soon as possible.

- Case 2.2: with Transfer Learning, using pre-trained model from Baseline

**Figure 19:**
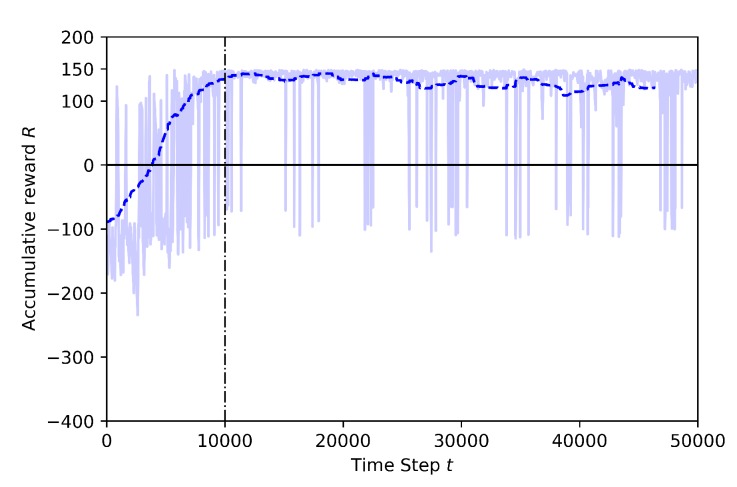
Training result for Case 2.2 by Transfer Learning.

In this training, the Baseline model, shown in Figure 11, is used as pre-trained to transfer to the learning drone the knowledge on how to catch the target drone. The last layer of the model is frozen and the other layers are trained. The training time is set to 50K steps and the annealing point to 10K steps. In Figure 19 the training results for transfer learning are shown. After the annealing point, as shown in Figure 18, the training with TL shows better results compared to the training without TL.

**Figure 20:**
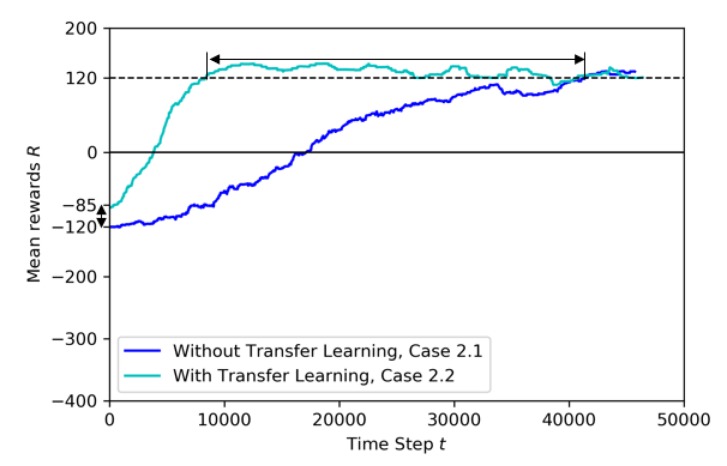
Training mean rewards for Case 2.1 and Case 2.2.

In Figure 20 the transfer learning metrics are shown in terms of jump-start and threshold time. TL starts training with mean reward around −85 while the training without TL is worse, around −120. The jump-start achieved is almost 35 more mean reward with transfer learning. Moreover, the TL also reaches a pre-specified performance level faster (at around 10K steps), while the model without TL reaches the threshold point just after 40K steps.

## 5. Further Model Results and Discussion

### 5.1. Comparison of the Effects of Different Annealing Points in TL

**Figure 21:**
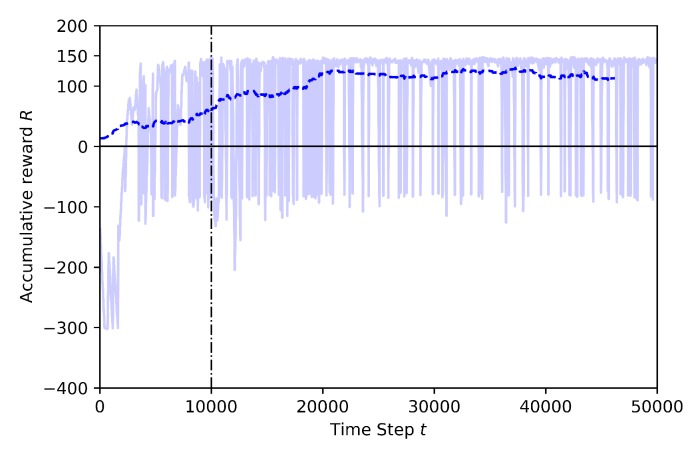
Training result for Case 1.2, Annealing at 10K steps.

**Figure 22:**
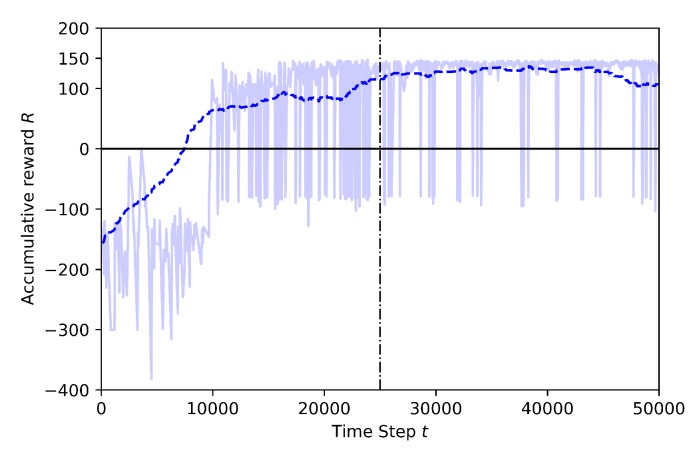
Training result for Case 1.2, Annealing at 25K steps.

Transfer learning training with different annealing lengths are compared for Case 1.2. The linear annealing policy is the same in all cases (from 1 to 0.1 randomness). However, different responses are found for different annealing points. For example, in Figure 21, it is seen that the annealing starts at 10K steps and the total training covers 50K steps. Before the annealing, the agent learns slowly and reaches higher rewards in 20K steps, but there are still crashed episodes. However, in Figure 22 the annealing is set at 25K steps. Although mean rewards are very low at the beginning of the training, after the agent explores more the environment, for around 15K steps, and it is able to reach high reward values. After the annealing, there are still crashes but the number of crashed episodes is lower than the crashed episodes compared to Figure 21.

**Figure 23:**
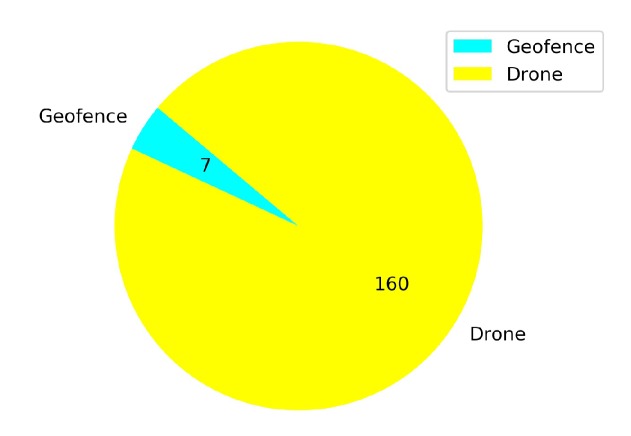
Crash report chart for Case 1.2, Annealing at 10K steps.

**Figure 24:**
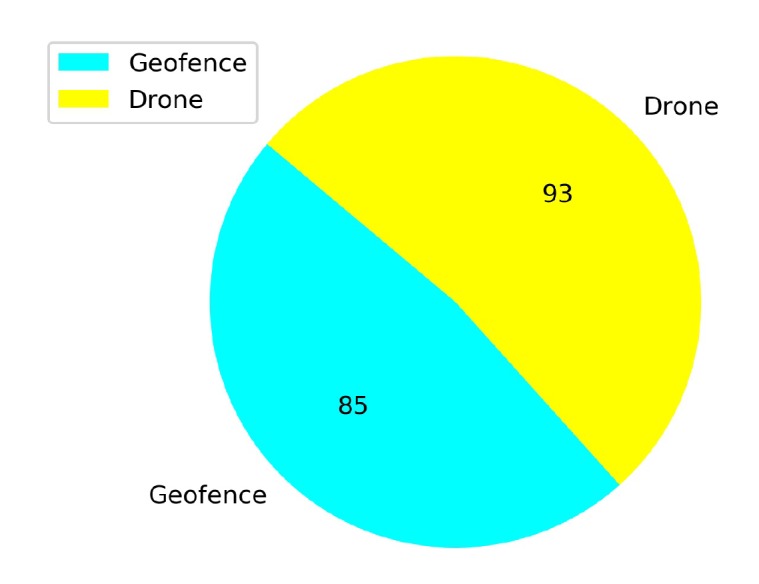
Crash report chart for Case 1.2, Annealing at 25K steps.

Figure 23 shows that from a total of 167 crashed episodes by annealing at 10K steps, only seven episodes crashed with the geofence while 160 episodes crashed with the stationary third drone. In Figure 24 the number of crashed episodes and their crashed obstacles are shown for annealing at 25K steps. There are 178 crashed episodes in total, 85 episodes crashed on geofence and 93 episodes crashed on drone. The total number of crashed episodes are slightly higher than before, but the number of episodes crashed on stationary drone are reduced in half. The main reason is that a longer annealing allows the agent to explore more at the beginning of the training, learning about both type of obstacles at the same time.

### 5.2. Comparison of the Explored Areas with or without TL

In this section, a map was built to show the drone positions during all the training steps. As in the previous comparison, the details of the crashed obstacles are also given. The map of the drone positions is limited by the coordinates of the geofence, these limits are [−5, 150] for the x-axis and [−70, 20] for the y-axis. The blue dots represent the agent position in each time step during training. The red dot is the initial position of the target drone.

**Figure 25:**
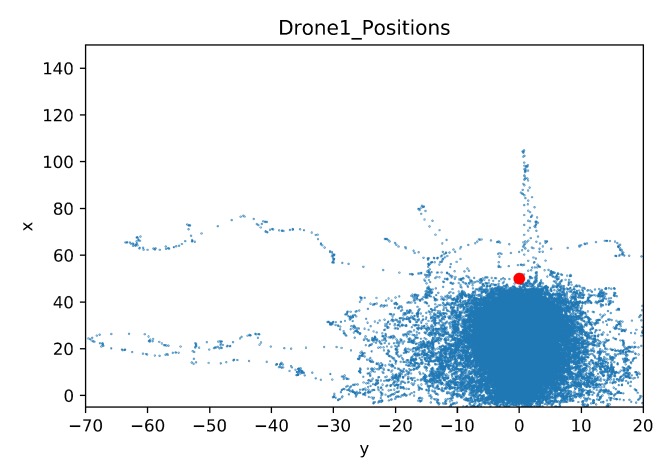
Drone Position Map for Baseline.

In Figure 25 the learning drone position map for Baseline is shown. It can easily be seen that the agent drone learns how to focus on targeting the goal, avoiding exploring areas that do not face the target. Also it is observed that geofence limits are not approached.

**Figure 26:**
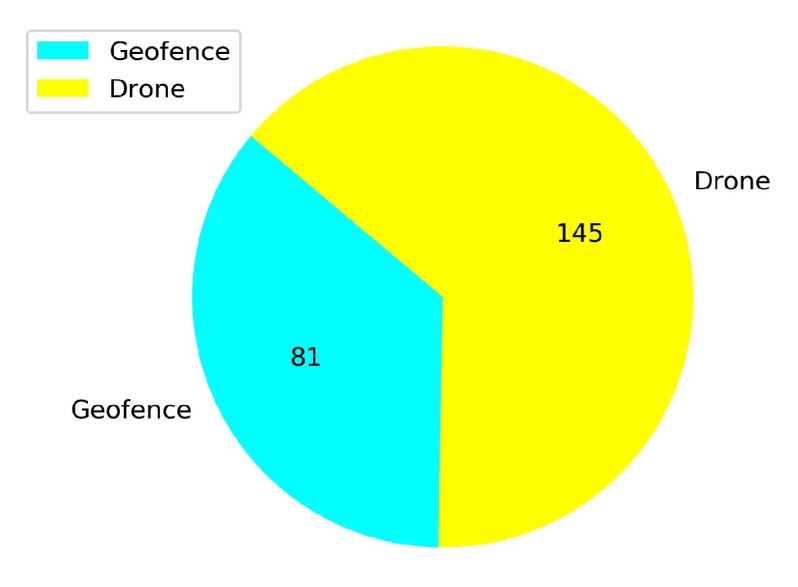
Crash report chart for Case 1.1.

In Figure 26 the number of total crashes and the crashed obstacles for the Case 1.1 are shown. As it is seen in Figure 26, there are 145 episodes crashed against the stationary drone. Moreover, it is observed that the number of crashes with the geofence are also higher in this training. The geofence limits are exceeded 81 times in this training, although it was 61 in the training without adding the stationary drone shown in Baseline.

**Figure 27:**
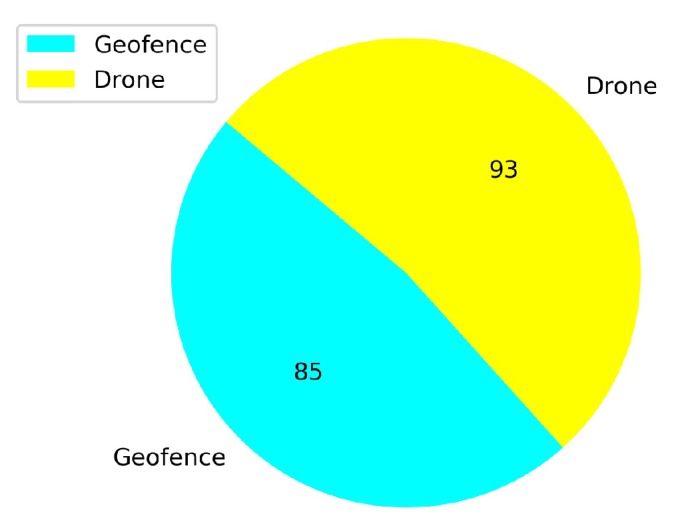
Crash report chart for Case 1.2 by Transfer Learning.

In Figure 27 the number of total crashes and the crashed obstacles for Case 1.2 are shown. As it is seen in Figure 27, there are 93 episodes crashed against the stationary drone. Moreover, it is observed that the number of crashes with the geofences is 85. The geofence limits are violated four times more with transfer learning compared with the full training of Case 1.1.

**Figure 28:**
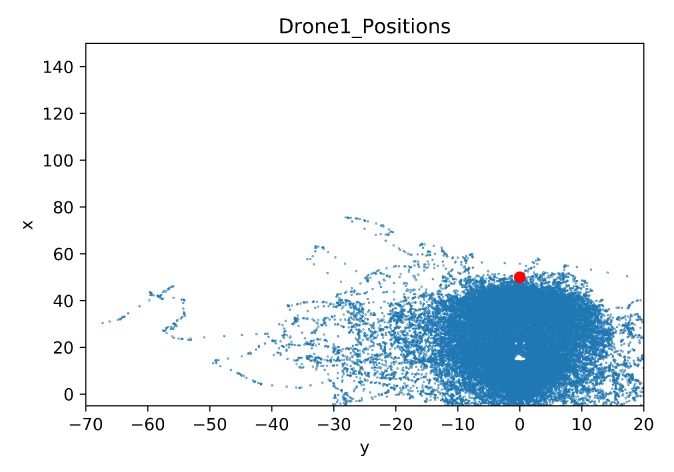
Drone Position Map for Case 1.1.

**Figure 29:**
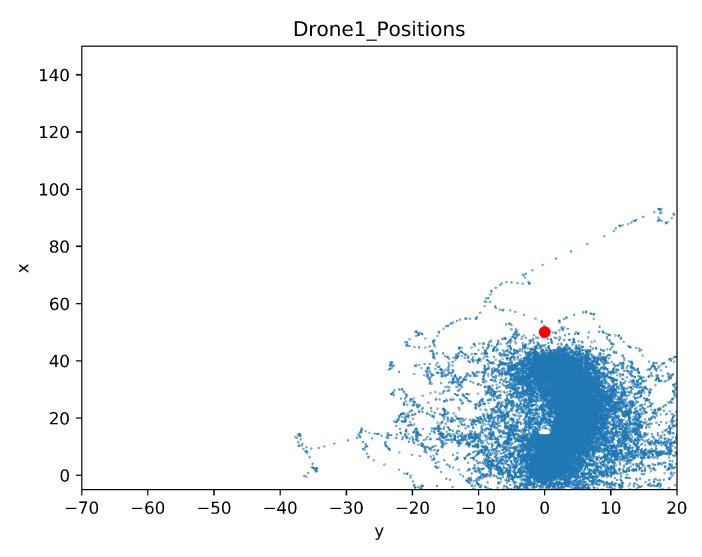
Drone Position Map for Case 1.2 by Transfer Learning.

Figure 28 and Figure 29 show maps of the learning drone position for Case 1.1 and Case 1.2 respectively. Observe that with transfer learning (Figure 29) the drone is mostly directed to the target, while without transfer learning, the drone is distracted and moves far away from the goal (see Figure 28).

**Figure 30:**
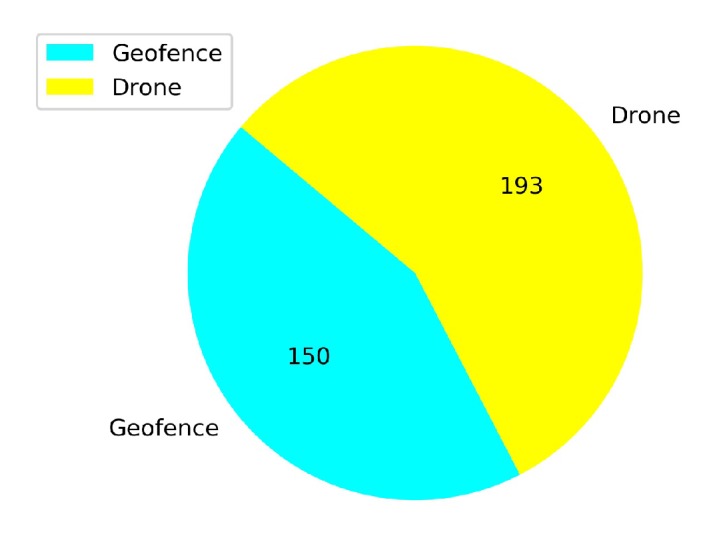
Crash report chart for Case 1.3.

In Figure 30 the number of the total crashes and the crashed obstacles for Case 1.3 are shown. There are more crashed episodes in Case 1.3 compared to the other cases because of the non-stationary drone. There are 193 episodes crashed this drone. Also, the number of episodes of crashing with the geofence (150) is high compared to the cases before. In summary, the learning drone does not learn how to avoid this non-stationary drone in a considerable time as expected in this case.

**Figure 31:**
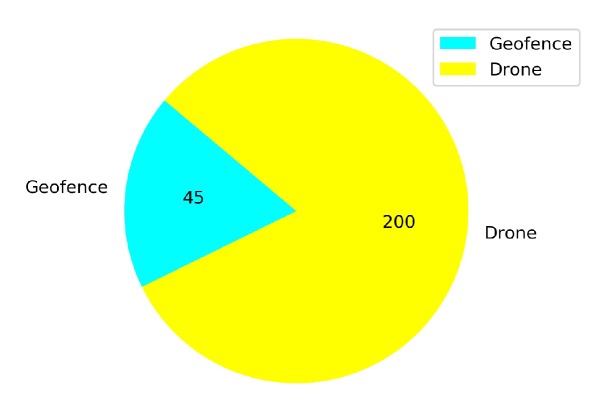
Crash report chart for Case 1.4 by Transfer Learning.

In Figure 31 the number of total crashes and the crashed obstacles for Case 1.4 are shown. A similar number of episodes failures (200) are also due to the non-stationary drone. However, the crashes with the geofence (45) is lower than in Figure 30 without transfer learning.

**Figure 32:**
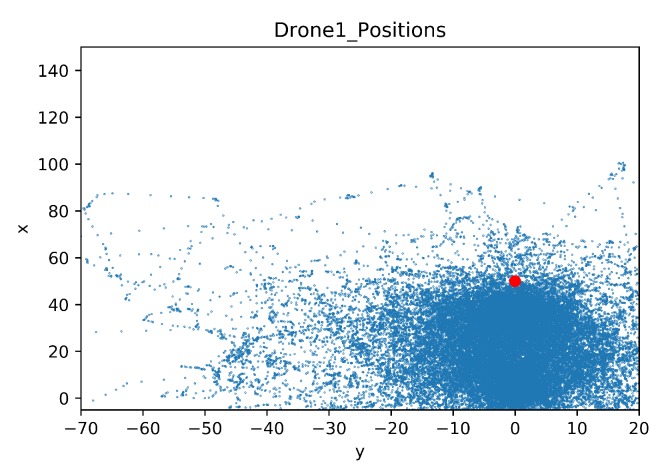
Drone Position Map for Case 1.3.

**Figure 33:**
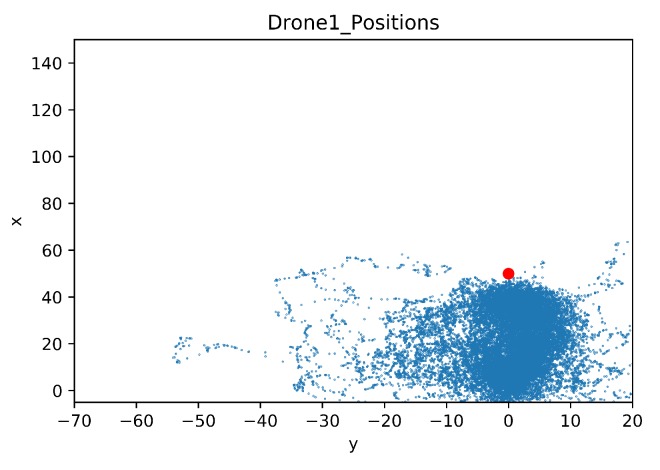
Drone Position Map for Case 1.4 by Transfer Learning.

In Figure 32 and Figure 33 the maps of the learning drone positions during the training sessions can be seen. With transfer learning (Figure 33) the drone is better focused on the target, while without transfer learning, the drone is moving left and right side of the environment in order to find a way to avoid non-stationary drone (Figure 32) but fails in reaching its goal.

**Figure 34:**
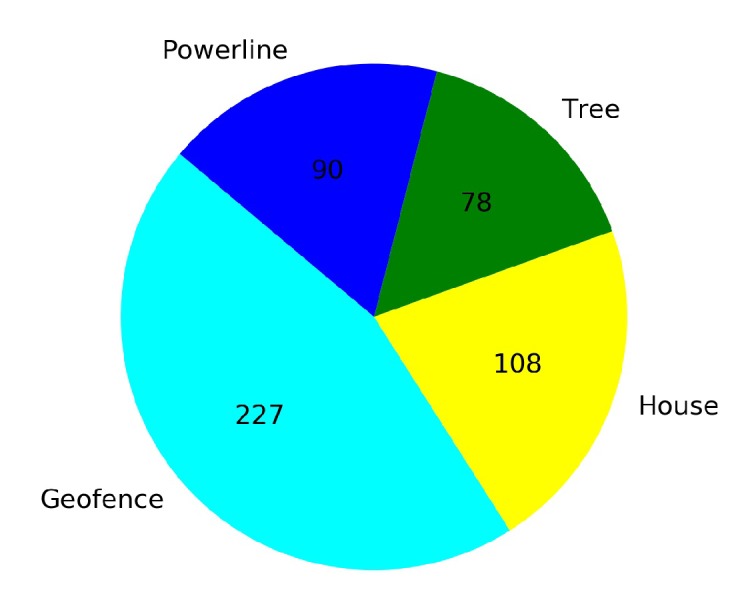
Crash report chart for Case 2.1.

**Figure 35:**
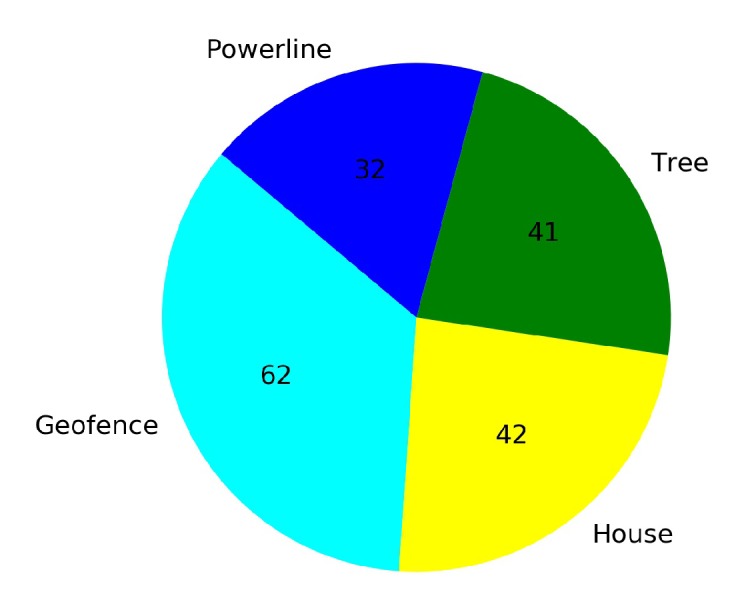
Crash report chart for Case 2.2 by Transfer Learning.

In Figure 34 and Figure 35 the number of total crashes and the crashed obstacles for Case 2.1 and Case 2.2 are presented respectively. Although the number of crashed episodes is 177 for the transfer learning Case 2.2, the number is even higher (503 crashed episodes) in Case 2.1. The total number of crashed episodes was reduced by 65% with transfer learning. The crashed obstacles are categorized as geofences, trees, power lines, and houses.

**Figure 36:**
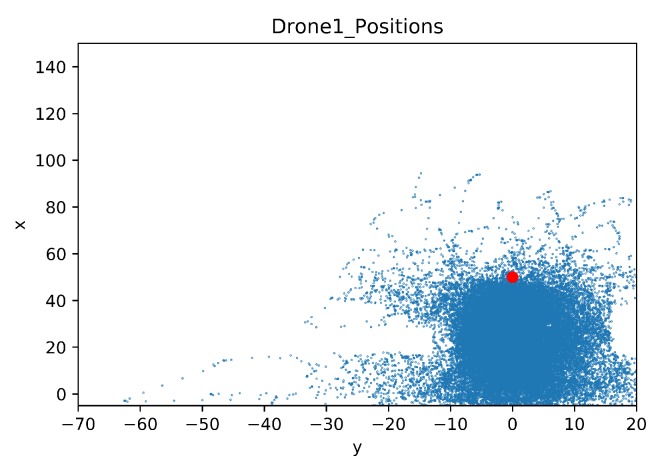
Drone Position Map for Case 2.1.

**Figure 37:**
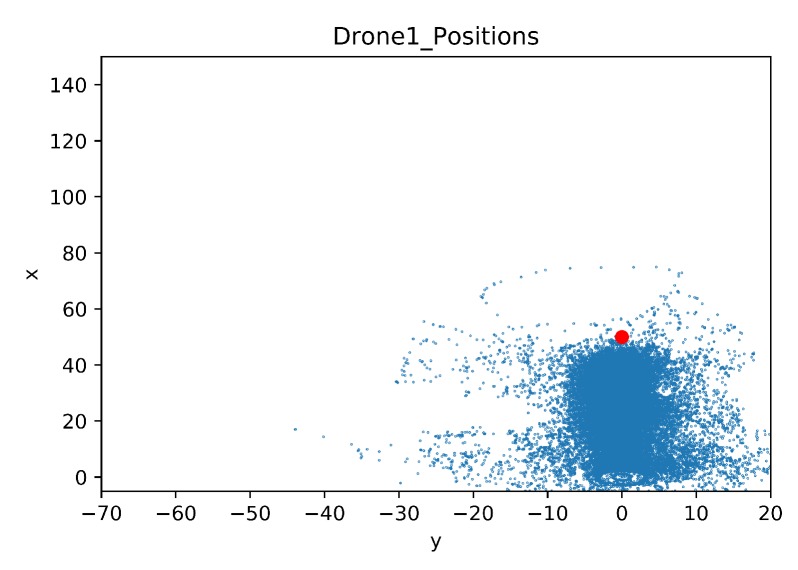
Drone Position Map for Case 2.2 by Transfer Learning.

In Figure 36 and Figure 37 the learner drone position maps in the environment during the training sessions are shown. In both figures, the shape of obstacles such as houses can be observed. For example, in Figure 36, the learner drone tries to explore the environment by moving around the house, as seen with a white rectangular shape. In addition, one of the trees (in front of the house) can also be seen as a white area surrounded by many blue dots. In Figure 37 the shape of the house can also be observed, but with transfer learning the learning drone does not need to explore all around the house.

### 5.3. Testing the Models at Low Altitude

In this section the tests results for Case 2.1 and Case 2.2, and are discussed. During a test, the agent is not learning anymore, but applies the learnt model with no more random behaviour. For this reason in this section we will not call the agent the learning drone, but the agent drone.

Each test set is made of 100 episodes, starting from the take-off and ending by catching the target drone (successful), by colliding (failure) or by time-out. Failures can happen by crashing with a visible ground obstacle (tree, house, wires, poles, etc.) or with the virtual geofence.

**Figure 38:**
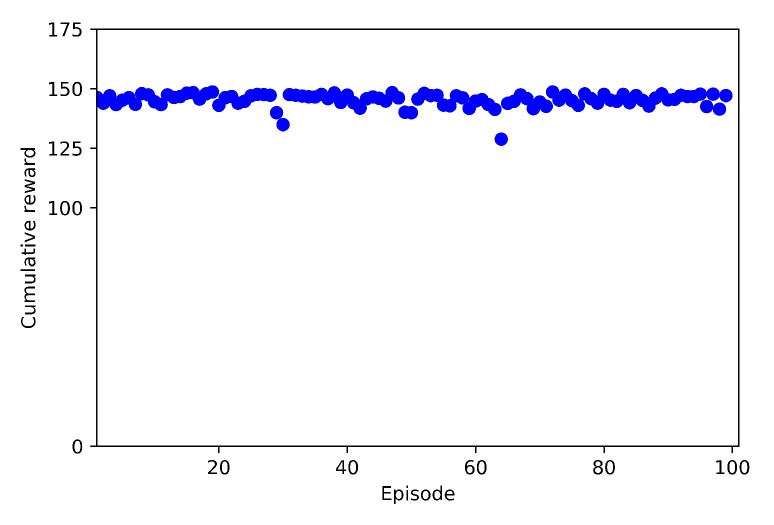
Test Result for Case 2.1 (Without TL).

**Figure 39:**
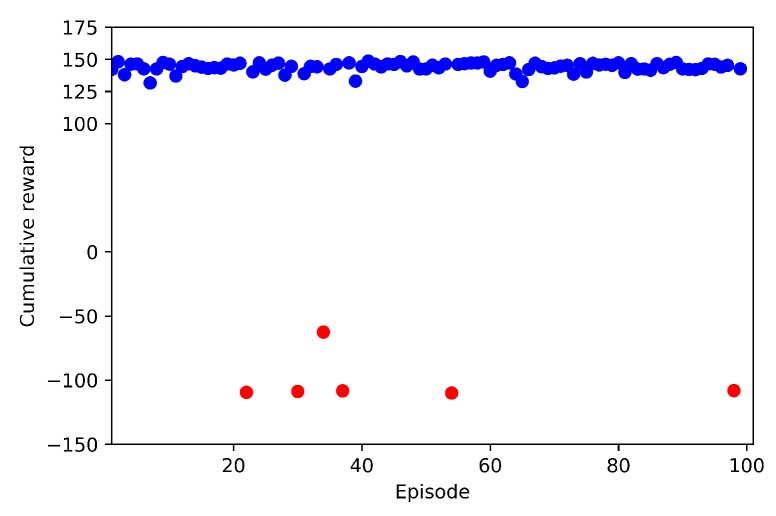
Test Result for Case 2.2 (With TL).

Test results are shown in Figure 38 for full training (Case 2.1), and in Figure 39 for transfer learning (Case 2.2). In all 100 episodes of Case 2.1, the agent drone was successful and able to catch the target drone without any crash. The cumulative reward plot is almost a straight line, with a few oscillations. On the contrary, with transfer learning (Case 2.2) the agent drone crashed 6 times out of 100 episodes. The main reason for these crashes is that the agent drone had learned only some of the obstacles in the environment, and thus, it missed some of the distant obstacles. However, even if there were failure episodes, the transfer learning showed a 94% success rate, which is good performance when considering the short time spent on training.

## 6. Conclusions

With the expansion of drones flying in the airspace, the availability of an effective counter-drone technology is a must. This counter-drone technology consists on several systems, on ground and on air, from which the solution presented in this paper is just one part. For this to work, it is necessary to have a support system that detects the target drone, classifies its activity as malicious, and estimates the position to the target drone. The Internet of Things solutions will be helpful in these tasks. The paper shows that deep reinforcement learning is a promising approach for the interception of the target drone moving randomly. However, the full system is still to be developed. In particular the final interception method, which is here achieved by crashing into the target drone, could use a more sophisticated approach to capture it.

The paper showed how the learning process can be improved by step-by-step learning. Initially, the drone learns the basic objective of its mission: head towards the target drone while moving inside an invisible geo-cage. Then, new secondary objectives can be further introduced using transfer learning. Our additional objectives were to avoid colliding with another (non-malicious) drone, or to avoid multiple but fixed obstacles (houses, trees, electrical wires, etc.). Transfer learning showed much better performance that starting a longer full training: It was faster in reaching a threshold reward and it did with a higher asymptotic performance.

Future improvements to the proposed method need to be made. In a future with many drones flying legally in the air, the correct discrimination of the target drone needs to be better ensured. The proposed agent’s state, with the front depth image and the target heading is too ambiguous when a third drone is in between the agent drone and the target drone. New ways to represent the state have to be tested. Moreover, the mix of fixed and movable obstacles has not yet been resolved. Finally, the very challenging 3D space needs to be addressed too.

Artificial intelligence improves itself very quickly and new methods and tools are being introduced at every moment. However, there is still little knowledge about how the predictions of artificial intelligence models work. In other words, it is not clear what makes them to choose the most convenient action. In the future, the methods for visualizing, explaining and interpreting deep reinforcement learning models need to be investigated [32]. Explainability of artificial intelligence is mandatory to address the certification of any avionics device. Moreover, artificial intelligence explainability can also help to improve the performance of the deep neural networks by detecting and eliminating parts of the state parameters that are not relevant.

## Figures and Tables

**Table 1:** Counter-drone techniques available according to [5].

Method Type	The Number of Cases Available
Jamming	96
Net	18
Spoofing	12
Laser	12
Machine Gun	3
Electromagnetic Pulse	2
Water Projector	1
Sacrificial Collision Drone	1
Other	6

**Table 2:** Rewards.

Reward	The Reason
+100	Goal reached
−100	Collision: Obstacle (stationary or moving) or geofence
−1 + Δ Distance + TrackAngle	Otherwise

**Table 3:** Training cases summary.

Case	Training	Steps	Annealing	Geofence	Obstacles
**Baseline**	FULL	125K	50K	YES	NONE
**Case 1.1**	FULL	75K	50K	YES	stationary 3rd drone
**Case 1.2**	Transferred	50K	25K	YES	stationary 3rd drone
**Case 1.3**	FULL	75K	50K	YES	non-stationary 3rd drone
**Case 1.4**	Transferred	50K	25K	YES	non-stationary 3rd drone
**Case 2.1**	FULL	125K	50K	YES	houses, trees, electrical, etc.
**Case 2.2**	Transferred	50K	10K	YES	houses, trees, electrical, etc.

## Abbreviations

The following abbreviations are used in this manuscript:

UAV	Unmanned Aerial Vehicle
AI	Artificial Intelligence
DRL	Deep Reinforcement Learning
CNN	Convolutional Neural Network
RL	Reinforcement Learning
TL	Transfer Learning
NED	North East Down
BVLOS	Beyond Visual Line of Sight
ApI	Application Programming Interface

## References

[1] European ATM Master Plan: Roadmap for the Safe Integration of Drones into All Classes of Airspace. https://www.sesarju.eu/node/2993.

[2] Fabra F., Zamora W., Sangüesa J., Calafate C.T., Cano J.C., Manzoni P. (2019). A Distributed Approach for Collision Avoidance between Multirotor UAVs Following Planned Missions. Sensors.

[3] Flights Diverted after Gatwick Airport. https://www.bbc.com/news/uk-england-sussex-48086013.

[4] Kratky M., Farlik J. (2018). Countering UAVs—The Mover of Research in Military Technology. Def. Sci. J..

[5] Michel A.H. (2018). Counter-Drone Systems; Center for the Study of the Drone at Bard College. https://dronecenter.bard.edu/counter-drone-systems.

[6] Akhloufi M.A., Arola S., Bonnet A. (2019). Drones Chasing Drones: Reinforcement Learning and Deep Search Area Proposal. Drones.

[7] Redmon J., Divvala S., Girshick R., Farhadi A. You only look once: Unified, real-time object detection. Proceedings of the IEEE Conference on Computer Vision and Pattern Recognition (CVPR).

[8] Anwar A., Raychowdhury A. (2019). Autonomous Navigation via Deep Reinforcement Learning for Resource Constraint Edge Nodes using Transfer Learning. arXiv.

[9] Unreal Engine 4. https://www.unrealengine.com/en-US/what-is-unreal-engine-4.

[10] Kouris A., Bouganis C.S. Learning to Fly by MySelf: A Self-Supervised CNN-based Approach for Autonomous Navigation. Proceedings of the 2018 IEEE/RSJ International Conference on Intelligent Robots and Systems (IROS).

[11] Lu X., Xiao L., Dai C., Dai H. (2018). UAV-aided cellular communications with deep reinforcement learning against jamming. arXiv.

[12] Rodriguez-Ramos A., Sampedro C., Bavle H., De La Puente P., Campoy P. (2019). A deep reinforcement learning strategy for UAV autonomous landing on a moving platform. J. Intell. Robot. Syst..

[13] Sutton R.S., Barto A.G. (1998). Reinforcement Learning: An Introduction.

[14] Kiumarsi B., Vamvoudakis K.G., Modares H., Lewis F.L. (2018). Optimal and autonomous control using reinforcement learning: A survey. IEEE Trans. Neural Netw. Learn. Syst..

[15] Mnih V., Kavukcuoglu K., Silver D., Rusu A.A., Veness J., Bellemare M.G., Graves A., Riedmiller M., Fidjeland A.K., Ostrovski G. (2015). Human-level control through deep reinforcement learning. Nature.

[16] Mnih V., Kavukcuoglu K., Silver D., Graves A., Antonoglou I., Wierstra D., Riedmiller M.A. (2013). Playing Atari with Deep Reinforcement Learning. arXiv.

[17] Van Hasselt H., Guez A., Silver D. Deep Reinforcement Learning with Double Q-Learning. Proceedings of the Thirtieth AAAI Conference on Artificial Intelligence.

[18] McClelland J.L., Mcnaughton B.L., O’Reilly R.C. (1995). Why there are complementary learning systems in the hippocampus and neocortex: Insights from the successes and failures of connectionist models of learning and memory. Psychol. Rev..

[19] Riedmiller M. Neural fitted Q iteration-first experiences with a data efficient neural reinforcement learning method. Proceedings of the 16th European Conference on Machine Learning.

[20] Lin L.J. (1993). Reinforcement Learning for Robots Using Neural Networks. Ph.D. Thesis.

[21] Hasselt H.V. Double Q-learning. Proceedings of the Advances in Neural Information Processing Systems 23: 24th Annual Conference on Neural Information Processing Systems 2010.

[22] Kersandt K. (2017). Deep Reinforcement Learning as Control Method for Autonomous UAVs. Master’s Thesis.

[23] Taylor M.E., Stone P. (2009). Transfer learning for reinforcement learning domains: A survey. J. Mach. Learn. Res..

[24] Shah S., Dey D., Lovett C., Kapoor A. (2017). AirSim: High-Fidelity Visual and Physical Simulation for Autonomous Vehicles. Field and Service Robotics.

[25] Brockman G., Cheung V., Pettersson L., Schneider J., Schulman J., Tang J., Zaremba W. OpenAI Gym 2016. https://arxiv.org/abs/1606.01540.

[26] Abadi M., Agarwal A., Barham P., Brevdo E., Chen Z., Citro C., Corrado G.S., Davis A., Dean J., Devin M. (2015). TensorFlow: Large-Scale Machine Learning on Heterogeneous Distributed Systems. arXiv.

[27] (2016). Theano Development Team. Theano: A Python framework for fast computation of mathematical expressions. arXiv.

[28] Plappert M. keras-rl. https://github.com/keras-rl/keras-rl.

[29] Von Bothmer F. (2018). Missing Man: Contextualising Legal Reviews for Autonomous Weapon Systems. Ph.D. Thesis.

[30] Gurriet T., Ciarletta L. Towards a generic and modular geofencing strategy for civilian UAVs. Proceedings of the 2016 International Conference on Unmanned Aircraft Systems (ICUAS).

[31] AirSim Documentation. https://microsoft.github.io/AirSim.

[32] Samek W., Wiegand T., Müller K.R. (2018). Explainable Artificial Intelligence: Understanding, Visualizing and Interpreting Deep Learning Models. arXiv.

